# The Effect of Binaural Beats Music-Based Interventions on the Autonomic Nervous System, Considering Kinesiophobia of Reinjuries and Fitness Biomarkers in Professional Athletes with Musculoskeletal Injuries: Study Protocol for a Double-Blind Randomized Controlled Trials

**DOI:** 10.3390/healthcare14142129

**Published:** 2026-07-15

**Authors:** Evangelos Kontogiannis, Marianna Papadopoulou, Dimitrios Mandalidis, Apostolos Z. Skouras, Maria Papandreou

**Affiliations:** 1 Laboratory of Advanced Physiotherapy, Physiotherapy Department, University of West Attika, 12243 Aigaleo, Greece; mpapad@uniwa.gr (M.P.); mpapand@uniwa.gr (M.P.); 2 Department of Neurology, School of Medicine, National and Kapodistrian University of Athens, Attikon University Hospital, 12462 Athens, Greece; 3 School of Physical Education and Sports Science, National and Kapodistrian University of Athens, 17237 Athens, Greece; dmndldis@phed.uoa.gr; 4 1st Department of Orthopaedic Surgery, National and Kapodistrian University of Athens, 12462 Athens, Greece; apostolis.sk@gmail.com

**Keywords:** binaural beats, kinesiophobia, autonomic nervous system, sympathetic skin response, VO_2_max, heart rate variability, open skills sports, lower-limb musculoskeletal injuries

## Abstract

**Background/Objectives:** This study aims to evaluate the effectiveness of binaural beats (BB) on the autonomic nervous system (ANS), kinesiophobia of reinjury and fitness biomarkers in professional athletes with chronic musculoskeletal injuries. **Methods**: A total of 54 athletes aged 18–25 years, engaged in open-skill sports and experiencing chronic lower-limb injuries for over three months, will be recruited. They will be randomly assigned to one of three groups: an intervention group, a placebo group, and a control group. All athletes will undergo a 15 min auditory intervention five times per week for four weeks. The intervention group will listen to BB embedded in music at high beta and gamma frequencies (30–100 Hz) with guided instructions. The placebo group will receive placebo BB within the same music framework, while the control group will listen only to the music background. Assessments will be conducted at baseline, immediately post-intervention, and at a 4-week follow-up. The primary outcomes will be kinesiophobia of reinjury, ANS function, and heart rate variability (HRV), assessed using the Tampa Scale of Kinesiophobia (TSK-17), Sympathetic Skin Response (SSR), and HRV analysis, respectively. Secondary outcomes will include sport-related anxiety and pain beliefs, assessed using the Sport Competition Anxiety Test (SCAT-15) and the Pain Beliefs and Perceptions Inventory (PBPI-16), respectively; handgrip strength, assessed using the Hand Grip Test; and fitness-related biomarkers, including maximal oxygen uptake (VO_2_max) and blood lactate concentration. **Conclusions**: These findings may provide evidence supporting BB as a complementary, non-invasive intervention to enhance psychological and physiological rehabilitation in injured athletes. Ethics and Dissemination: The study has received ethical approval and is registered on ClinicalTrials.gov. Findings will be disseminated through peer-reviewed publications, conferences, and academic presentations.

## 1. Introduction

Musculoskeletal injuries (MSK-I) represent the most prevalent type of injury among athletes and are associated with substantial physical, psychological, and social consequences that can delay or compromise return to sport (RTS) [1]. The etiology of MSK-I is multifactorial, involving a complex interaction between intrinsic factors—such as sex, age, previous injury history, neuromuscular imbalances, and psychological traits—and extrinsic factors, including training load, technical execution, playing surface, equipment, and environmental conditions [2,3,4]. Epidemiological studies consistently demonstrate that professional athletes sustain injuries more frequently than amateur athletes, largely due to higher training volumes, increased physical and energetic demands, and intensified competitive schedules [5,6].

Injury mechanisms vary according to sport-specific demands, with contact actions (e.g., tackling) and complex, high-speed, multi-joint movement patterns (e.g., throwing while jumping or cutting maneuvers) being among the most common contributors [7,8]. Consequently, both injury incidence and anatomical distribution differ considerably across sports disciplines. Athletes participating in open-skill sports such as football, volleyball, basketball, etc., characterized by unpredictable and externally driven environments, exhibit higher injury rates compared with those engaged in closed-skill sports such as swimming, tennis, martial arts, etc., which involve more stable and predictable movement contexts [9]. For example, professional football reports approximately 8.1 musculoskeletal injuries focused on lower limbs per 1000 exposure hours, whereas swimming reports around 1.54 injuries per 1000 h [10,11]. A recent systematic review indicated that athletes participating in open-skill sports (e.g., basketball, handball, and volleyball) exhibit a higher incidence of lower-limb injuries compared to injuries in other body regions. The most frequently reported injuries in these athletes involve the knee and ankle [12].

Considering the distinct characteristics and demands of open-skill versus closed-skill sports, the present study focused on open-skill athletes because these sports, such as football, basketball, handball, and volleyball, are characterized by unpredictable movement environments, rapid decision-making, opponent interaction, and frequent cutting, jumping, and landing actions. All these sports are associated with an increased risk of lower-limb injury [9]. The heightened cognitive demands inherent to open-skill sports may further elevate injury risk, as athletes are required to continuously engage attentional control, decision-making, and cognitive flexibility, particularly under conditions of fatigue or competitive pressure [13]. These factors may compromise optimal motor execution and neuromuscular control during sport-specific tasks. Accordingly, fear of reinjury is particularly relevant in open-skill athletes during the return-to-sport process, as psychological readiness may directly influence movement confidence, functional performance, and safe reintegration into competitive activity.

Recent consensus-based recommendations in sports injury rehabilitation emphasize that return-to-sport decision-making should be considered a multidimensional and biopsychosocial process, integrating physical recovery, psychological readiness, contextual factors, and risk-management considerations [13,14]. Within this framework, interventions targeting fear of reinjury, autonomic regulation, and psychophysiological readiness may be clinically relevant adjuncts to conventional rehabilitation approaches.

Return to Sports (RTS) process is influenced by an intricate interplay of physical recovery, psychological readiness, and social context [15,16]. Notably, a substantial proportion of athletes experience reinjury upon returning to training or competition, highlighting the limitations of approaches that focus exclusively on physical rehabilitation [17]. Among the biopsychosocial factors affecting RTS outcomes, kinesiophobia has emerged as a critical determinant. A recent systematic review identified kinesiophobia as one of the most influential psychological consequences of sports injuries, significantly contributing to reinjury risk and negatively affecting physical performance, emotional state, and self-confidence [18].

Kinesiophobia is defined as an excessive, irrational fear of movement or physical activity stemming from a perceived vulnerability to pain or reinjury [18]. Elevated levels of kinesiophobia have been positively associated with anxiety, depressive symptoms, attentional disturbances, and reduced self-efficacy during RTS [19,20]. In athletes following anterior cruciate ligament (ACL) reconstruction, kinesiophobia—alongside pain, anxiety, depression, and low self-esteem—has been shown to impair RTS and increase reinjury likelihood across multiple sports, including football, basketball, handball, and rugby [17,19]. Similarly, chronic ankle instability has been linked to heightened kinesiophobia and diminished functional capacity in athletic populations [21]. The Tampa Scale of Kinesiophobia (TSK) is widely used to quantify fear of movement in injured athletes and has demonstrated strong psychometric properties across sports and clinical contexts [22].

Beyond psychological factors, MSK-has been shown to influence central and peripheral nervous system functioning, particularly the Autonomic Nervous System (ANS). Emotional and cognitive responses to injury can modulate ANS activity, shifting the balance between sympathetic (“fight-or-flight”) and parasympathetic (“rest-and-digest”) responses [23]. Cardiovascular regulation, vasomotor activity, and electrodermal responses represent key physiological pathways through which emotional stress and pain-related cognitions manifest following injury [24]. Heart rate variability (HRV) and sympathetic skin response are widely recognized as sensitive indicators of ANS function and autonomic balance [24]. Studies demonstrated that the Isometric Hand Grip Test (IHG) can be a supplementary test to assess autonomic nervous system regulation through metrics the changes in heart rate and blood pressure during implementation is isometric contraction at 30% MVC [25,26].

Emerging evidence suggests that pain intensity, pain catastrophizing, and perceived disability following injury and chronic pain are closely associated with alterations in HRV, reflecting dysregulation of autonomic control [27,28]. Conversely, findings in elite football players indicate a transient increase in HRV approximately 72 h post-injury, suggesting enhanced parasympathetic activation during early recovery [29]. Despite these insights, the relationship between ANS function and kinesiophobia in injured athletes remains largely unexplored.

Hand Grip Strength (HGS) performance elicits acute ANS activation leading to increases in heart rate, blood pressure, and respiratory rate [30,31]. Hand grip test performance may be indirectly influenced by fear, anxiety, or pain anticipation, particularly in athletes recovering from injury, potentially confounding performance outcomes [32]. Recent studies have highlighted a potential link between handgrip strength and mental health outcomes, suggesting that HGS may reflect psychological well-being [33,34]. Liyan, [33] assessed that higher handgrip strength appears to be associated with a lower likelihood of developing generalized anxiety and depressive disorders. Priyadharsan et al, [34] assessed the effect of examination stress on skeletal muscle function among first-year medical students using the HGS test and demonstrated that Maximum Voluntary Contraction (MVC) and endurance time were significantly reduced during the stress state compared to the relaxation state. Despite these insights, the relationship between stress on skeletal muscle function and kinesiophobia in professional athletes with previous lower-limb injuries remains largely unexplored.

Contemporary rehabilitation approaches increasingly emphasize the integration of physical and psychological interventions to address the multidimensional consequences of sports injuries. Programs combining therapeutic exercise, cognitive–behavioral strategies, and pain neuroscience education have demonstrated efficacy in reducing kinesiophobia and improving functional outcomes [35]. Mind–body interventions have been shown to enhance mindfulness, pain resilience, and emotional regulation, thereby supporting RTS [35]. Additionally, psychosomatic interventions such as music or acoustic signal interventions have been proposed as promising strategies to alleviate kinesiophobia and enhance athletic performance [18].

Acoustic signals or music-based interventions delivered through auditory stimuli, stimulating the auditory nerve and activating the brain’s cortex, can be applied during training or prior to competition. They have been shown to positively influence psychophysiological states by modulating emotional, cognitive, behavioral, and physiological responses [36,37]. In athletes, music-based interventions have been associated with improvements in aerobic and anaerobic capacity, as well as enhanced cardiovascular and hormonal regulation, increased motivation, better emotional control, and reduced perceived exertion and fatigue [38,39,40]. However, the effects of acoustic signals or music-based interventions such as binaural beats on kinesiophobia and reinjury prevention following sports-related MSK-I remain insufficiently investigated.

Binaural beats (BB) are an auditory phenomenon often layered with background music and referred to as binaural music. BB occurs when two tones of slightly different frequencies are presented separately to each ear. The brain then perceives a third tone, known as a binaural beat, which equals the difference between the two frequencies. For instance, if one ear hears a tone at 300 Hz and the other at 306 Hz, the brain detects a binaural beat of 6 Hz [41]. Binaural beats require the use of headphones so that each frequency reaches only one ear. The sound of binaural beats is created by the brain rather than by the physical mixing of sound waves in the air; they are perceived binaurally and are often experienced as stronger, associated with the induction of specific brainwave states [41]. BB have gained attention for their potential psychological and physiological effects, particularly in stress modulation and attentional control by using different frequencies (such as delta, theta, beta, alpha and gamma waves). In athletic contexts, binaural beats have been suggested to reduce pre-competition anxiety and enhance concentration, which may be especially beneficial during rehabilitation and RTS [42,43,44]. Physiologically, binaural beats appear to influence ANS activity by modulating sympathetic–parasympathetic balance. Listening to theta-frequency binaural beats post-exercise has been shown to increase HRV, indicating enhanced parasympathetic activation and relaxation [45]. Similar findings have been reported in stress-recovery contexts among military personnel [46]. Evidence examining the effects of binaural beats on kinesiophobia, ANS activity, reinjury anxiety, pain beliefs, and fitness biomarkers in athletes with chronic musculoskeletal injuries remains limited.

Binaural beats constitute a non-invasive form of auditory stimulation that may modulate athletes’ psychophysiological state by influencing cortical oscillatory activity, arousal regulation, and ANS function [41,44,45]. This mechanism is particularly relevant to kinesiophobia, which extends beyond maladaptive beliefs to encompass anxiety, heightened threat perception, increased physiological arousal, and ANS dysregulation. Consequently, binaural beats may reduce fear-related responses by promoting autonomic balance, enhancing psychological readiness, and supporting confident movement.

Athletes who participate in open-skill sports were selected because these sports involve unpredictable movement environments, which may increase the risk of lower-limb injury. Therefore, interventions that improve autonomic regulation, such as binaural beats, may help reduce fear of reinjury.

Therefore, based on this theoretical and clinical reasoning, the present study aims to investigate the effects of binaural beat stimulation on kinesiophobia of reinjury, on ANS function through the Sympathetic Skin Response (SSR) and the Isometric Hand Grip test (IHG) and the fitness biomarkers such as VO_2_max, blood lactate concentration, and HRV in professional athletes with chronic lower-limb musculoskeletal injuries (MSK-I). A secondary objective is to examine the maintenance of these effects four weeks after the intervention. It is hypothesized that binaural beats favorably modulate ANS activity, reduce kinesiophobia, reinjury anxiety, and pain beliefs, and enhance aerobic capacity, with sustained effects at follow-up. Conversely, the null hypothesis posits that binaural beat stimulation will not significantly affect ANS function, psychological outcomes, or fitness biomarkers, nor will any effects be maintained after four weeks.

## 2. Materials and Methods

### 2.1. Participants

A total of 54 professional male and female athletes aged 18–25 years will be recruited from collaborating sports clubs located in the Attica region of Greece. Eligible participants will be athletes participating in open-skill sports, including football, basketball, volleyball, and handball, who have sustained at least one chronic lower-limb musculoskeletal injury lasting more than three months. Recruitment will be conducted in collaboration with team coaches and sports medicine professionals affiliated with these clubs. Participants will be randomly assigned in a 1:1:1 ratio to one of three groups: the experimental group, the placebo group, or the control group (*n* = 18 per group). Randomization will be performed using a computer-generated random allocation sequence by an independent researcher who is not involved in participant recruitment, outcome assessment, or statistical analysis. Allocation concealment will be ensured using sequentially numbered, opaque, sealed envelopes. Participants, outcome assessors, and statisticians will remain blinded to group allocation throughout the study.

Sample size was calculated using G*Power software (version 3.1.9.7; Heinrich Heine University Düsseldorf, Düsseldorf, Germany) [47]. The calculation was based on a mixed-design repeated-measures ANOVA with three groups (experimental, placebo, and control) and three measurement time points (baseline, post-intervention, and four-week follow-up). Assuming a medium effect size (f = 0.25), an alpha level of 0.05, statistical power of 0.95 and equal allocation across groups, the minimum required sample size was estimated at 48 participants. To account for an anticipated dropout rate of approximately 10%, the final target sample size was set at 54 participants, with 18 participants allocated to each group. A medium effect size of Cohen’s f = 0.25 was selected as a realistic estimate for a non-invasive psychophysiological intervention (BB) targeting psychological and Performance outcomes. This estimate was considered appropriate because previous intervention studies examining kinesiophobia and rehabilitation-related outcomes in athletes with lower-limb injuries have reported comparable sample sizes (33–51 athletes) and medium effect estimates (0.25–0.40) [48,49,50].

To qualify for the study, participants must be diagnosed with at least one musculoskeletal injury (≥3 months) by a sports medicine doctor with at least 5 years of experience and their injury must have been repaired. Athletes must be active members of their respective team with a frequency of engaging in sports 3–5 times per week, including both training sessions and competitions. Additionally, they must be referred to as having kinesiophobia with TSK scores ranging from 17 to 37. Exclusion criteria encompassed hearing impairments, recent lower-limb injuries, recent surgery or a history of concussion. All inclusion and exclusion criteria for athletes to be recruited in the study are described in Table 1.

All measurements will be conducted at the Laboratory of Sports Excellence, the Orthopedic Research & Education Center of the University General Hospital “ATTIKON. Ethical approval was granted by the University of West Attica’s Ethics Committee (Approval No. 62664), adhering to the Declaration of Helsinki, and all participants will provide written informed consent before enrolment. Additionally, the protocol of this study was registered in the www.clinicaltrials.gov database (accessed on 17 January 2026) under identifier NCT07370636.

### 2.2. Procedures

**Figure 1:**
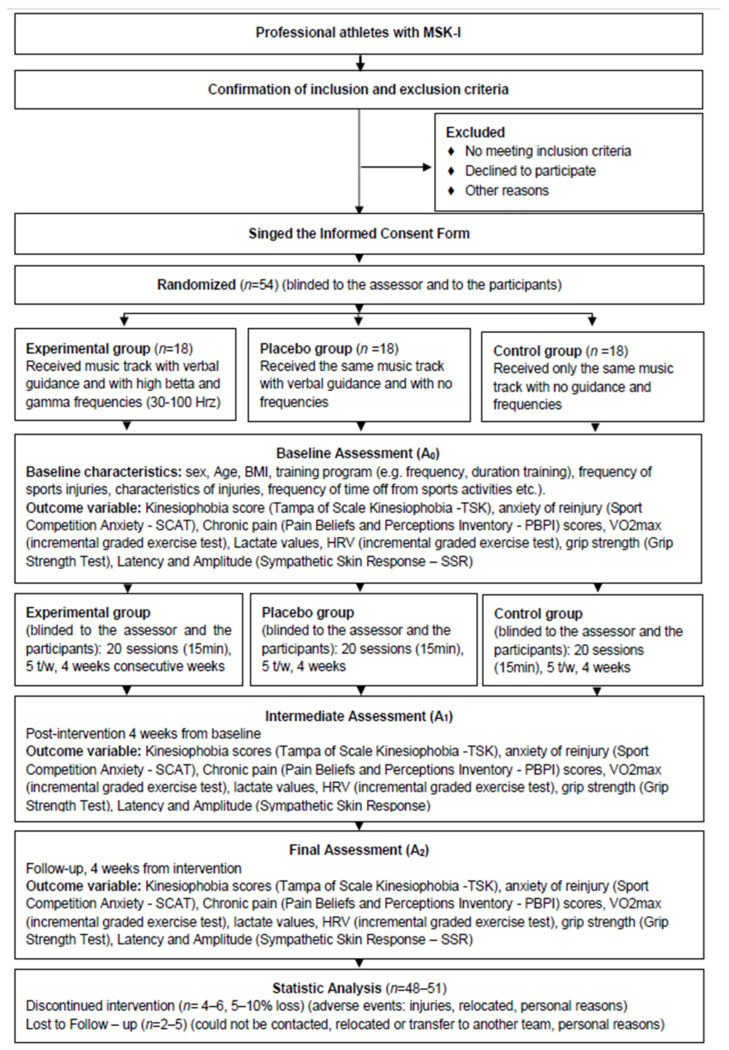
CONSORT Flow diagram.

This double-blind randomized controlled trial will investigate the effects of binaural beats on biopsychological outcomes in professional athletes with a history of chronic lower-limb musculoskeletal injuries. The study will be conducted in three phases: baseline (pre-intervention), immediately after the four-week intervention, and four weeks after completion of the intervention. Outcome measures will include self-report questionnaires assessing kinesiophobia, sport-related anxiety, and pain beliefs; physiological assessments of autonomic nervous system (ANS) function; handgrip strength; and fitness-related biomarkers, including maximal oxygen uptake (VO_2_max), blood lactate concentration, and heart rate variability (HRV). All athletes will complete a four-week standardized binaural beats music-based intervention incorporated into the warm-up phase prior to each training session and official game. The BB intervention will be administered 5 times per week (pre-training and pre-game), resulting in a total of 20 sessions. Each session will consist of 15 min of music listening (Figure 1).

Prior to initiating the intervention, athletes will receive detailed oral and written information regarding the study aims and assessment procedures from licensed physiotherapists with five years of clinical research experience. Data collection will include both subjective and objective measures and will be conducted at three time points: baseline (pre-intervention), immediately post-intervention, and four weeks after the completion of the intervention. Written informed consent will be obtained from all participants before enrollment.

### 2.3. Main Outcome Measures

All questionnaires will be administered in three phases: baseline (pre-intervention), immediately after the four-week intervention, and four weeks after completion of the intervention for all three groups. The questionnaires will assess kinesiophobia, sport-related anxiety, and pain-related beliefs. Final questionnaire scores will be used for statistical analysis, and predefined scoring criteria will be applied where relevant to characterize participants’ baseline psychological status.

#### 2.3.1. Tampa Scale of Kinesiophobia Questionnaire

The Tampa Scale of Kinesiophobia (TSK-17) is a self-report questionnaire designed to assess pain-related fear, which is considered a key component in the cognitive evaluation of musculoskeletal pain within the biopsychosocial model. The TSK consists of 17 items distributed across two dimensions: (i) activity avoidance, based on the belief that physical activity may lead to reinjury, and (ii) somatic focus [50,51]. Each item is rated on a 4-point Likert scale ranging from 1 (strongly disagree) to 4 (strongly agree). The total score is calculated after reversing the score (4 → 1) for items 4, 8, 12, and 16, resulting in a possible score range from 17 to 68 [52]. Total scores ≤ 37 indicate low levels of kinesiophobia, whereas scores > 37 indicate high levels [53]. The Greek version of the TSK-17 has been validated in the Greek population, demonstrating satisfactory internal consistency (Cronbach’s α = 0.74) and good test–retest reliability (ICC = 0.78) in individuals with chronic low back pain [54].

#### 2.3.2. Sport Competition Anxiety Test

The Sport Competition Anxiety Test (SCAT-15) is a self-report questionnaire designed to assess competitive anxiety in athletes [55]. The SCAT consists of 15 items related to various psychological factors such as anxiety, concentration and decision-making in training or competition settings [56]. Each item is rated on a 3-point Likert scale ranging from 1 to 3. The total score ranges between 10 and 30 points. Scores below 17 indicate a low level of competitive anxiety, scores between 17 and 24 indicate a moderate level of competitive anxiety, while scores above 24 indicate a high level of competitive anxiety. The Greek version of the SCAT-15 has been validated in the Greek population, demonstrating high internal consistency (Cronbach’s α = 0.85–0.87) and test–retest reliability (ICC = 0.84–0.89) [57].

#### 2.3.3. Pain Beliefs and Perceptions Inventory

The Pain Beliefs and Perceptions Inventory (PBPI) is a self-report questionnaire designed to identify cognitive and behavioral factors that influence pain management and athletic performance. The PBPI is a widely used, comprehensive, and psychometrically sound instrument that is brief and easy to administer. It consists of 16 items distributed across three subscales: (i) Time, (ii) Mystery, and (iii) Self-Blame [58]. Each item is rated on a 5-point Likert scale ranging from −2 to +2. The total score is calculated after reversing the score (2 → −2) for items 3, 9, 12, and 15, yielding a possible score range from −32 to +32 [59]. Higher total scores indicate more negative beliefs about pain and its management, which are associated with increased levels of anxiety, depression, disability, and pain severity [60]. The Greek version of the PBPI-16 has been validated in the Greek population, demonstrating high internal consistency (Cronbach’s α = 0.89–0.96) and good test–retest reliability (ICC = 0.73–0.82) among individuals with chronic pain [59].

#### 2.3.4. Sympathetic Skin Response: Autonomic Nervous System Function

Autonomic nervous system function will be evaluated through Sympathetic Skin Response (SSR) and through grip strength in two phases: pre-intervention and 4 weeks post–BB intervention for all three groups. Additionally, these procedures will be repeated during the follow-up period.

**Figure 2:**
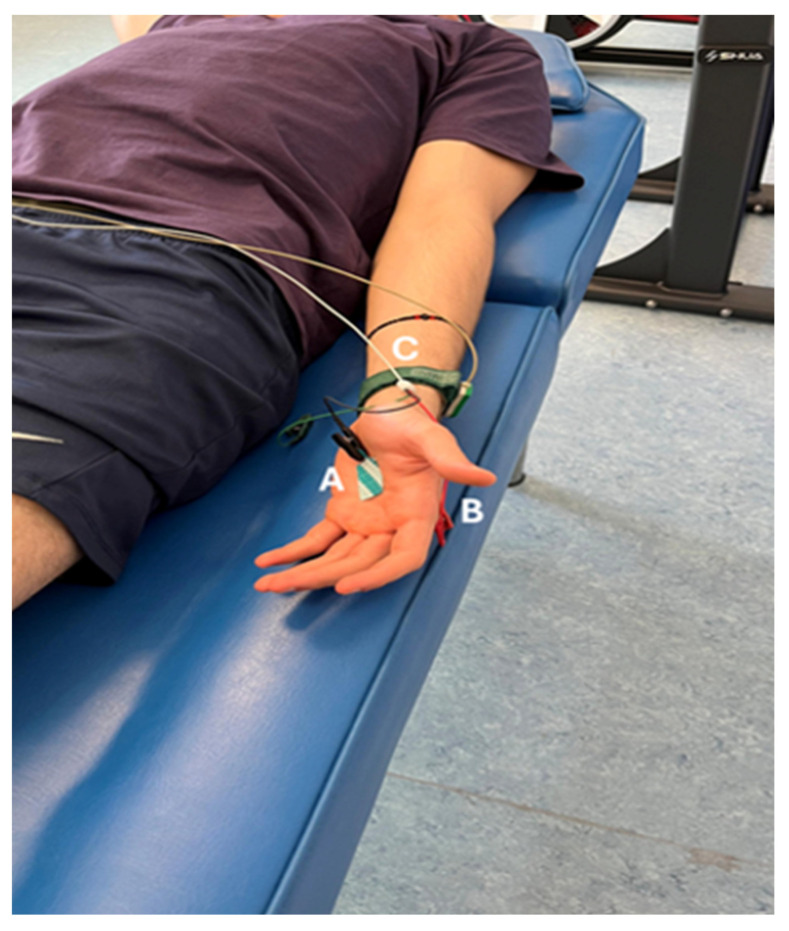
Electrode placement for Sympathetic Skin Response (SSR) recording from the upper limb. Surface electrodes were positioned according to the standard SSR recording protocol. (A) Active recording electrode placed on the palmar surface of the hand, (B) reference electrode positioned on the dorsal aspect of the hand, and (C) ground electrode placed on the distal forearm. This electrode configuration was used for all upper-limb SSR recordings throughout the study [62].

**Figure 3:**
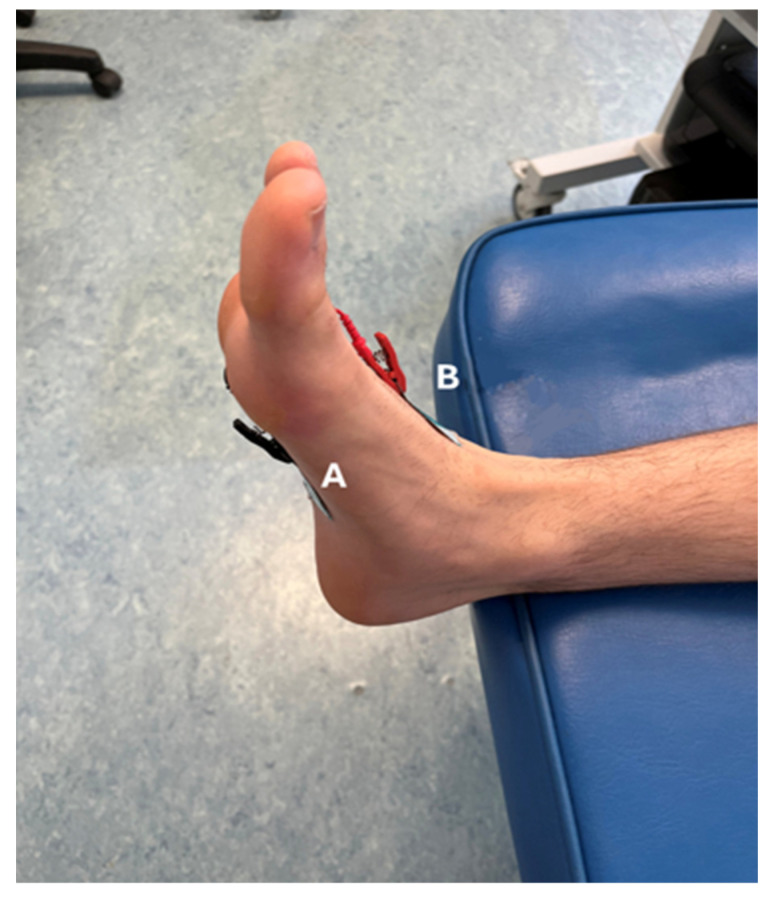
Electrode placement for Sympathetic Skin Response (SSR) recording from the lower limb. Surface electrodes were positioned according to the standardized SSR recording protocol. (A) Active recording electrode placed on the plantar surface of the foot (sole), and (B) reference electrode positioned on the dorsal aspect of the foot. This electrode configuration was used for all lower-limb SSR recordings performed throughout the study to evaluate sudomotor sympathetic function [62].

**Figure 4:**
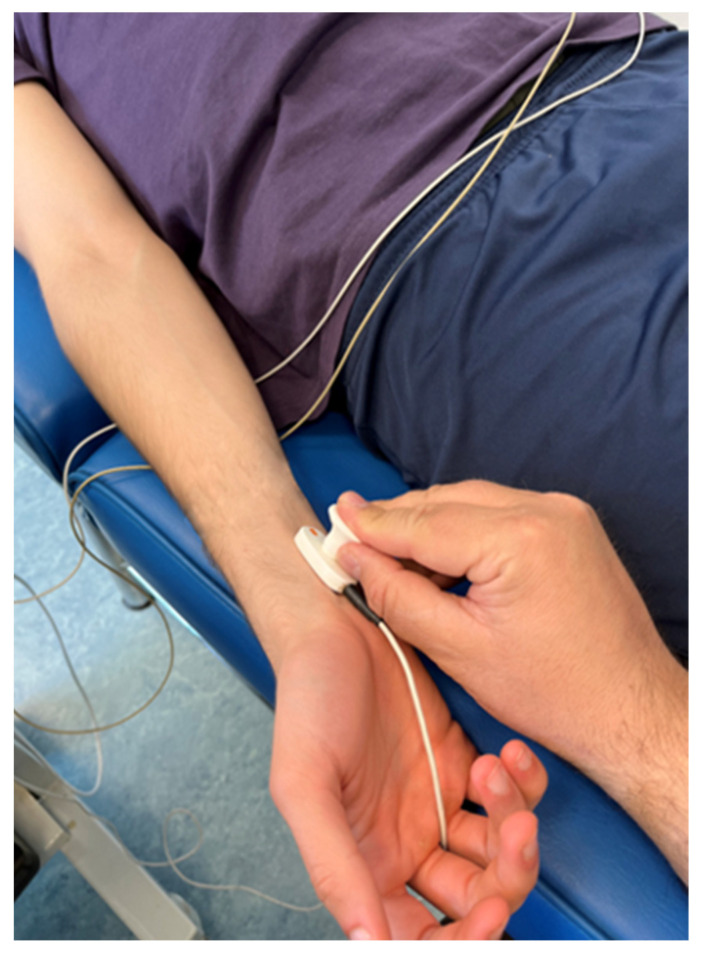
Electrical stimulation site for Sympathetic Skin Response (SSR) recording. The median nerve at the wrist was stimulated using surface stimulating electrodes to evoke the sympathetic skin response. Electrical stimulation was delivered according to the standardized neurophysiological protocol (75 mA, 0.1 ms duration), with stimuli administered at random interstimulus intervals of at least 30 s to minimize habituation and ensure reliable SSR recordings [62].

SSR is a neurophysiological tool designed to assess the functional integrity of the sympathetic cholinergic sudomotor pathways. It is a non-invasive, routine test used to assess autonomic dysfunction, specifically measuring how sweat glands (sudomotor activity) respond to various stimulus, such as electrical or auditory stimulation. SSR testing will be conducted using the Dantec Keypoint system (version 5.13; Alpine Biomed ApS, Skovlunde, Denmark), following the standard protocol [61]. All athletes will be instructed to refrain from smoking and to consume only a light breakfast, excluding alcohol and caffeine. The examination room temperature will be maintained at 23–26 °C, and limb temperature at 32–33 °C. Recordings will be obtained using surface, circular electrodes. The active electrodes will be positioned on the left palm and right sole, while the reference electrodes will be placed on the dorsum of the corresponding hand and foot. Electrical stimuli will be delivered to the right wrist at an intensity of 75 mA and a duration of 0.1 ms. The band-pass filter will be set between 0.5 and 2000 Hz. Five stimuli will be administered unpredictably at random intervals of at least 30 s. Latency will be measured from the onset of the stimulus artifact to the first deflection from baseline (in seconds), and amplitude will be measured peak-to-peak (from negative to positive peak, in millivolts). The shortest latency and the highest amplitude among the five responses will be used for statistical analysis. Parameters included in the analysis will be: SSR upper-limb latency, SSR upper-limb amplitude, SSR lower-limb latency, and SSR lower-limb amplitude (Figure 2, Figure 3 and Figure 4).

#### 2.3.5. K-Force Dynamometer: Hand Grip Strength

Hand Grip Strength Test (HGS) is a widely used method for assessing grip strength through the performance of a maximal voluntary contraction (MVC) [63]. The HGS has demonstrated high test–retest reliability (ICCs = 0.90–0.99) in evaluating grip force during maximal isometric contractions [64]. In the present study, MVC will be recorded using the K-Force Controller (KFC) portable dynamometer (Kinvent Biomécanique SAS, Montpellier, France). Data will be transmitted via Bluetooth Low Energy (2.4 GHz) to the Kinvent software (version 2.24.1; Kinvent Biomécanique SAS, Montpellier, France), where they will be stored and exported to external data storage devices. The K-Force Grip dynamometer has been shown to have excellent reliability (ICC = 0.96–0.97) in recording grip strength [65]. For the test procedure, participants will be seated with the shoulder in a neutral position, the elbow flexed at 90° without support, the forearm in a neutral position, and the wrist positioned between 0° and 30° of dorsiflexion and 0–15° of ulnar deviation. After MVC assessment, participants will be instructed to squeeze the dynamometer handle at an intensity corresponding to at least 30% of their MVC, a threshold known to elicit autonomic nervous system activation. Participants will perform three trials, each lasting 30 s, with 1 min rest intervals between trials. The test will be performed bilaterally, beginning with the dominant upper limb, using identical positioning and testing procedures for both sides.

#### 2.3.6. Ergospirometry Test: Fitness Biomarkers

An ergospirometry test will be used to evaluate the physical fitness biomarkers such as maximal oxygen uptake (VO_2_max) and blood lactate concentration (REF). All these measurements will be conducted in two phases: pre-intervention and 4 weeks post–BB intervention for all three groups. Additionally, these procedures will be repeated during the follow-up period.

##### Maximal Oxygen Uptake and Lactate Levels

The ergospirometry test will be conducted on a treadmill following the protocol described by Bielik et al. [66]. Respiratory variables will be recorded using a breath-by-breath gas analyzer (K5, Cosmed, Rome, Italy), calibrated prior to each session according to the manufacturer’s instructions. Maximal oxygen uptake (VO_2_max) will be evaluated as an objective measure of aerobic capacity and as a robust indicator of cardiorespiratory function [67]. VO_2_max will be determined via an incremental graded exercise test (GXT), which progressively increases treadmill velocity until volitional exhaustion, with estimated time of at least 12–20 min [68].

Before the start of the test, the researchers will record HR and blood pressure in a resting state. Athletes will be placed in a supine, relaxed position and instructed to remain still and refrain from speaking for 5 min. Prior to starting the test, all athletes will perform a standardized warm-up comprising 5 min of cycling on a cycle ergometer followed by 5 min of dynamic lower-limb stretching.

The blood lactate concentrations (La) will be measured in two phases before and at the end of the ergospirometry test and the results (mmol) will be used for statistical analysis.

The GXT will begin at 8 km·h^−1^, with velocity increments of 1 km·h^−1^ per minute until volitional exhaustion, maintaining a 0% treadmill gradient. Termination criteria will include (a) a plateau or decline in VO_2_ despite increasing workload, accompanied by a respiratory exchange ratio ≥ 1.10, (b) the ratio VE/VO2 ≥ 30, (c) the ratings of perceived exertion (RPE) 18–20 of borg scale and (d) the concentration of lactic acid in blood ≥ 9 mmol/L [67]. Athletes will receive verbal encouragement, primarily during the final minute. Respiratory variables will be continuously recorded using a breath-by-breath gas analyzer, which will be calibrated prior to each session according to the manufacturer’s instructions. VO_2_ values will be averaged over the final 10 s of each 1 min stage. All procedures will be supervised by two exercise physiologists to ensure participant safety and protocol fidelity. The results of oxygen uptake relative to body mass (VO_2_/kg), the metabolic equivalents (METs) and the ratio of carbon dioxide production to oxygen consumption (VCO_2_/VO_2_), which is defined as respiratory quotient (RQ), will be used for statistical analysis.

##### Kubios Software: Heart Rate Variability

Kubios software will be used to evaluate heart rate variability (HRV). HRV is a recognized indicator for assessing the activity of the autonomic nervous system (ANS) in the regulation of cardiovascular function and its adaptive response to various stimuli [69]. HRV has been shown to be a reliable measure (ICC 0.78–0.90) for evaluating cardiovascular performance and autonomic function in athletes [70]. In the current study, HRV recording will be carried out using a Polar H10 telemetric heart rate monitor (Polar Electro Oy, Kempele, Finland). Data analysis will be performed with the Kubios HRV software (version 1.8.14; Kubios Oy, Kuopio, Finland). HRV will be determined via an incremental graded exercise test (GXT), which progressively increased treadmill velocity until volitional exhaustion. The GXT will begin at 8 km·h^−1^, with velocity increments of 1 km·h^−1^ per minute until volitional exhaustion, maintaining a 0% treadmill gradient. Heart rate (HR) will be recorded across three distinct phases: pre-exercise (resting phase), during exercise, and post-exercise (recovery phase).

Prior to commencing the test, resting heart rate variability (HRV) will be recorded for five minutes in a supine position. The examiners will ask the athletes not to talk or move during the recording of heart rate variability. All athletes will follow a standardized 10 min warm-up protocol, consisting of 5 min of aerobic exercise and 5 min of stretching, primarily targeting the muscles of the lower limbs. Subsequently, they will undergo a graded treadmill exercise test, commencing at a speed of 8 km·h^−1^, with incremental increases of 1 km·h^−1^ per minute until volitional exhaustion is reached. Immediately after test completion, participants will be placed in supine position, remaining still and refraining from speaking again, and the examiners will record the HRV of recovery for 15 min. Recovery HRV will then be recorded in three phases: 0–5 min, 5–10 min, and 10–15 min. Blood lactic acid will be recorded before and after exercise, and all athletes must have a level ≥ 2 mmol/L before starting the test.

For the assessment of HRV, time-domain indices will be used, including the interval between two successive heartbeats (RR interval), the number of heartbeats per unit of time (HR), the standard deviation of normal-to-normal intervals (SDNN), and the root mean square of successive differences between adjacent intervals (RMSSD). In addition, frequency-domain measures will be analyzed, including the absolute power of heart rate variability in the low-frequency band (LF: 0.04–0.15 Hz) and the high-frequency band (HF: 0.15–0.40 Hz), the ratio of low- to high-frequency power (LF/HF), and total power (TP) [71,72]. For the statistical analysis, mean values of RR intervals (s), mean heart rate (HR; bpm), SDNN (ms), RMSSD (ms), LF (ms^2^), HF (ms^2^), LF/HF ratio (%), and total power (TP; ms^2^) will be used, as recorded during the resting phase and across the three phases of the recovery phase.

### 2.4. Intervention: The Binaural Beats Intervention Program

BB is delivered through an auditory stimulus waveform that is interpreted as a brainwave pattern by the Reticular Activating System (RAS). The RAS regulates arousal, kinesiophobia, attention, and awareness—key elements of consciousness itself [73,74]. In this study, BB is layered with background music and will be provided in MP3 format following specific guidance, in the English language, based on fearless motion. All stimuli will be presented binaurally at High Beta and Gamma frequencies, via an auditory system incorporating headphones [75]. In fact, the purpose of High Beta and Gamma frequencies on athletes is to improve the athlete’s psychomotor and self-regulation ability, their confidence, and subsequent performance in important competitions [76]. The Binaural Beats (BB) intervention program will be designed and produced by the Monroe Institute (Faber, VA, USA).

This BB music-based intervention will be received in high beta and gamma frequencies (30–100 Hertz) at 30 bpm (slow tempo), 60 bpm (normal tempo), and 100 bpm (fast tempo). The binaural beat stimuli will consist of mixed sinusoidal tones producing frequency patterns will crop in length to a duration of six times intervals (total 15 min): 0–2 min the slow tempo (Grounding), 3 min (2–5 min) the slow tempo (Relaxation/Intention Setting), 3 min (5–8 min) the normal tempo (Imagery Begins), 2.5 min (8–10.5 min) normal tempo (Emotional Processing + Imagery/Movement), 2.5 min (10.5–13 min) fast tempo (Active Visualization + Affirmation), and 2 min (13–16 min) fast tempo (Return to Awareness, Light Activation) enabling athletes to hear the music; this duration will also be likely to yield more pronounced emotional response [77].

The intensity will be modified at approximately the isochrone tones to 14–16 dB.

Each athlete will receive the musical intervention via the website binaukine.com, which has been specifically designed by the researchers exclusively for the purposes of the present study. Furthermore, participants’ compliance with the study protocol will be monitored by the researchers through the frequency of the athletes’ visits to the website.

The BB music-based intervention will be performed prior to each training session or competition, as part of the athletes’ warm-up routine. Athletes will be instructed to conduct the listening sessions in a quiet environment, seated comfortably with their eyes closed, and to avoid distractions from external stimuli during the intervention. All groups will receive the BB music-based interventions for 20 sessions, lasting 15 min per session, 5 times per week, over a period of 4 weeks.

Before beginning the BB music-based intervention phase, all athletes will attend a familiarization session during which the researchers will provide detailed instructions on how to perform the intervention. During the main intervention phase, the first session will be supervised by the research team, while the remaining sessions will be carried out individually, at different times according to each athlete’s training schedule. Each group will randomly receive the BB music-based intervention program.

The intervention group will receive a complete BB music-based intervention program. The placebo group will receive the same music-based intervention program with the verbal guidance but without the binaural beats. The control group will listen only to the music track, with the same music background as the intervention and placebo groups.

### 2.5. Statistical Analysis

Data will be summarized using descriptive statistical methods. Continuous variables will be presented as means and standard deviations, whereas categorical variables will be expressed as frequencies and percentages. Normality of continuous variables will be assessed using the Shapiro–Wilk test.

Baseline demographic, anthropometric, and clinical characteristics will be compared across the three study groups to assess baseline comparability. Continuous variables will be analyzed using one-way analysis of variance (ANOVA) or the Kruskal–Wallis test, as appropriate, whereas categorical variables will be compared using Pearson’s chi-square test or Fisher’s exact test. Ordinal variables will be analyzed using the chi-square test for trend where applicable. If clinically meaningful baseline imbalances are detected, adjusted analyses using mixed-design ANCOVA models will be conducted.

The primary analysis will follow the intention-to-treat principle, whereby all randomized participants will be analyzed in the groups to which they were originally allocated, regardless of adherence to the intervention protocol or study completion. Missing data will be examined before analysis by assessing the extent and pattern of missingness. Where appropriate, missing values will be handled using multiple imputation procedures, and sensitivity analyses will be performed to evaluate the robustness of the findings.

To investigate changes across the 4-week BB intervention period (Time factor) and differences among the three groups (experimental, placebo, and control), a mixed-design analysis of variance (ANOVA) will be performed. The assumptions of the mixed-design repeated-measures ANOVA, including normality of the model residuals, homogeneity of variances, and sphericity, will be assessed before analysis. This analysis will assess the main effects of Time (within-subjects factor) and Group (between-subjects factor), as well as their interaction [78]. When significant effects are identified, post hoc pairwise comparisons will be performed with appropriate adjustment for multiple comparisons. If clinically meaningful baseline imbalances are detected, adjusted analyses using mixed-design ANCOVA models will be conducted.

The clinical relevance of the findings was determined using partial eta squared (η^2^) as an index of effect size. Effect sizes were interpreted as small (η^2^ ≥ 0.01), moderate (η^2^ ≥ 0.06), or large (η^2^ ≥ 0.14) [67]. For continuous outcomes, estimated mean differences, standardized effect sizes, exact *p*-values, and corresponding 95% confidence intervals (95% CI) will be reported as appropriate. For categorical outcomes, odds ratios with 95% CI will be reported where applicable. All statistical analyses will be performed using SPSS version 29.0, with statistical significance set at *p* < 0.05.

## 3. Discussion

Lower-limb musculoskeletal injuries (MSK-I) are highly prevalent among professional athletes participating in open-skill sports and are associated with impaired return to sport (RTS), recurrent injury, reduced athletic performance, and persistent psychological distress [14,15,16,17]. Among the psychological factors influencing RTS, kinesiophobia has been identified as an important determinant of reinjury anxiety, movement avoidance, reduced self-confidence, impaired concentration, and decreased athletic performance [17,18]. Fear of reinjury and pain-related anxiety may also affect autonomic nervous system (ANS) regulation by increasing psychophysiological arousal, sympathetic activation, and altered cardiovascular responses.

The present study is based on a biopsychophysiological framework linking kinesiophobia, ANS regulation, and auditory stimulation using binaural beats (BB). Changes in skin electrical activity and heart rate variability (HRV) following BB stimulation may help characterize physiological responses associated with kinesiophobia, fear of reinjury, and related physiological biomarkers. In this context, changes in skin electrical activity, assessed using the Sympathetic Skin Response (SSR), reflect sympathetic ANS activity, whereas HRV provides information regarding cardiac autonomic regulation. Therefore, the simultaneous assessment of psychological outcomes (e.g., kinesiophobia, fear avoidance, anxiety, and pain beliefs), HRV, handgrip strength, and fitness-related biomarkers (VO_2_max and blood lactate accumulation) may provide a multidimensional evaluation of the interaction between psychological and physiological factors in athletes participating in open-skill sports with chronic lower-limb MSK-I.

Contemporary sports rehabilitation increasingly supports the integration of complementary psychophysiological interventions alongside conventional physical rehabilitation protocols. Acoustic stimulation interventions, including binaural beats (BB), have attracted growing scientific interest because of their potential effects on anxiety regulation, pain perception, attentional control, autonomic modulation, and psychophysiological performance [37,38,39,40,41,42,43,45]. Previous studies suggest that BB stimulation may reduce anxiety, improve attention, modulate pain perception, and influence ANS function [40,41,43,45].

However, limited evidence exists regarding the effects of BB in professional athletes with chronic lower-limb MSK-I, particularly with respect to kinesiophobia, autonomic function, handgrip strength, HRV, and fitness-related biomarkers. The present study therefore aims to address this gap by investigating whether a music-based binaural beats intervention can influence psychological and physiological outcomes relevant to fear of reinjury. It is hypothesized that reductions in kinesiophobia, competitive anxiety, and maladaptive pain beliefs will be accompanied by improvements in ANS function, as reflected by SSR parameters (latency and amplitude) and HRV following BB stimulation. Improvements in autonomic regulation may subsequently enhance cardiovascular recovery, exercise tolerance, handgrip strength, and aerobic fitness biomarkers, thereby contributing to a safer and more confident RTS process.

## 4. Practical Applications

If the intervention proves effective, binaural beats music-based stimulation may represent a non-invasive, low-cost, and easily applicable complementary strategy for sports rehabilitation. Such an intervention could be integrated into warm-up routines, pre-training preparation, or functional rehabilitation sessions in professional athletes with chronic lower-limb musculoskeletal injuries.

From a clinical perspective, this approach may assist physiotherapists, sports medicine physicians, athletic trainers, psychologists, and rehabilitation specialists in addressing psychological and physiological barriers to RTS. Binaural beats may help reduce stress-related arousal, support autonomic regulation, enhance movement confidence, and improve psychological readiness in athletes experiencing fear of reinjury. In addition, adding acoustic stimulation during warm-up and rehabilitation may provide useful information regarding its potential role in supporting attentional control, concentration, decision-making, and sport-specific performance.

The findings of this study may therefore contribute to the development of evidence-based complementary rehabilitation strategies aimed at reducing kinesiophobia, supporting ANS regulation, and facilitating safer reintegration into open skills sports.

## 5. Strengths and Limitations

A major strength of the present study is its double-blind randomized controlled trial design, which minimizes selection and assessment bias and enhances methodological rigor. The inclusion of experimental, placebo, and control groups allows the specific effects of binaural beats to be examined beyond the potential effects of music exposure and verbal guidance alone. In addition, the simultaneous evaluation of psychological outcomes, ANS function, handgrip strength, cardiovascular regulation (HRV), and fitness biomarkers provides a comprehensive biopsychophysiological assessment of professional athletes in open skill sports with chronic MSK-I. The inclusion of follow-up measurements four weeks after completion of the intervention further enables the examination of short-term maintenance effects.

Another strength of the study is the standardization of the intervention protocol. The binaural beats music-based intervention will be administered through headphones to ensure accurate binaural auditory stimulation. Furthermore, intervention duration, listening frequency, warm-up integration, auditory guidance, and environmental instructions will be standardized across participants to reduce variability in auditory exposure and improve intervention fidelity. The use of validated questionnaires and standardized physiological and functional assessment procedures further strengthens the reliability of the outcome measures and the internal validity of this study.

Nevertheless, several limitations should be acknowledged. First, participants will be recruited from collaborating sports clubs within the Attica region, Greece, which may introduce selection bias and limit the generalizability of the findings to athletes from other geographical regions, competitive levels, or sporting disciplines. Second, the study focuses exclusively on chronic lower-limb MSK-I, restricting the applicability of the findings to acute injuries, upper-limb musculoskeletal conditions, or post-surgical populations.

Additional limitations include heterogeneity in injury characteristics, such as injury duration, severity, diagnosis, rehabilitation history, training load, and competitive level, all of which may influence recovery and intervention responsiveness. Although the inclusion of both male and female athletes enhances the generalizability of the study, the sample is not specifically powered to examine sex-specific effects. Therefore, any sex-related differences in psychological, autonomic, or fitness-related responses will be interpreted with caution and considered exploratory. Finally, environmental listening conditions during independently completed intervention sessions may not be fully controlled, potentially introducing variability in participants’ exposure to the auditory intervention.

## 6. Conclusions

This study protocol describes a double-blind randomized controlled trial designed to investigate the effects of a binaural beats music-based intervention on kinesiophobia of reinjury, autonomic nervous system function, and fitness biomarkers in professional athletes with chronic lower-limb musculoskeletal injuries. By integrating psychological, autonomic, functional, and fitness-related outcomes, the study may provide a comprehensive understanding of how auditory stimulation could influence biophysiological functions.

The findings of the present study may contribute to the development of complementary, non-invasive rehabilitation strategies aimed at optimizing both psychological and physiological recovery in injured athletes. Future research should investigate whether the effects of binaural beat–based interventions differ between athletes participating in open-skill and closed-skill sports, particularly regarding kinesiophobia, autonomic nervous system regulation, cognitive performance, and physical fitness outcomes. In addition, further randomized controlled trials are warranted to evaluate the effectiveness of binaural beat interventions following surgical rehabilitation procedures, such as anterior cruciate ligament reconstruction and shoulder reconstruction, with particular emphasis on return-to-sport (RTS) outcomes, fear of reinjury, psychological readiness, and functional recovery. Finally, future studies should incorporate electroencephalography (EEG) alongside autonomic and psychological assessments to further elucidate the neurophysiological mechanisms underlying binaural beat stimulation and its potential role in enhancing rehabilitation outcomes in injured athletes.

## Figures and Tables

**Table 1:** Inclusion and exclusion criteria for athlete eligibility in the present study.

Inclusion Criteria	Exclusion Criteria
Professional athletes participate in open-skill sports (e.g., football, basketball, volleyball, handball).	Amateur or recreational athletes.
Male and female athletes aged 18–25 years.	Athletes are younger than 18 years or older than 25 years.
Participation in national and/or international professional championships for ≥5 years.	Athletes participate in closed-skill sports (e.g., tennis, martial arts, swimming).
History of lower-limb musculoskeletal injury sustained ≥3 months before enrollment.	Current musculoskeletal injury (<3 months) or recent orthopedic surgery.
Previous lower-limb musculoskeletal injury without complete functional rehabilitation.	Athletes who have fully recovered and completed rehabilitation following previous lower-limb musculoskeletal injuries.
Presence of low-to-moderate kinesiophobia, defined as a Tampa Scale for Kinesiophobia (TSK-17) score ranging from 17 to 37.	Poorly managed rehabilitation, ongoing medication affecting study outcomes, or recent surgery.
Presence of competitive anxiety, defined as a Sport Competition Anxiety Test (SCAT) score >17.	Musculoskeletal injuries sustained during the off-season period.
Regular participation in training and competitions 3–5 times per week.	History of concussion sustained during training or competition.
Adequate English-language proficiency to understand study procedures and complete the questionnaires.	Presence of neurological disorders, psychiatric disorders, or psychological conditions that could interfere with participation.
	Hearing impairment or inadequate English-language proficiency preventing completion of the study procedures.

TSK-17 = Tampa Scale for Kinesiophobia (17-item version); SCAT = Sport Competition Anxiety Test.

## Data Availability

The data presented in this study will be made available upon reasonable request from the corresponding author, due to ethical and confidentiality constraints. The dataset contains sensitive personal information collected with informed consent, and public disclosure is not permitted by the ethics committee. The raw Excel dataset can be provided upon reasonable request.

## Abbreviations

MSK-I	Multidisciplinary Digital Publishing Institute
RTS	Return To Sports
ACL	Anterior Cruciate Ligament
TSK	Tampa Scale of Kinesiophobia
ANS	Autonomic Nervous System
HRV	Heart rate variability
HGS	Hand Grip Strength
VO_2_max	Maximal Oxygen uptake
SCAT	Sport Competition Anxiety
PBPI	Pain Beliefs and Perceptions Inventory
SSR	Sympathetic skin response
GXT	Graded Exercise Test
HR	Heart Rate
La	blood Lactate concentration
RPE	Ratings of Perceived Exertion
SDRR	Standard Deviation of RR interval
SDNN	Standard Deviation of Normal-to-Normal intervals
RMSSD	Root Mean Square of Successive Differences
LF	Low Frequency power
HF	High Frequency power
LF/HF	Low Frequency to High Frequency ratio
TP	Total Power
MVC	Maximal Voluntary Contraction
nLF	normalized Low-Frequency
nHF	normalized High-Frequency
